# Digital *versus* manual workflows for cataract surgery: a systematic review and meta-analysis

**DOI:** 10.31744/einstein_journal/2025RW1478

**Published:** 2025-08-25

**Authors:** Dillan Cunha Amaral, Laura Cheidde, Denisse J. Mora-Paez, Eduardo Hissa Haddad, Pedro Lucas Machado Magalhães, Lucas Macedo Nascimento, Isabelle Rodrigues Menezes, Edson dos Santos-Neto, Mário Luiz Ribeiro Monteiro, Ricardo Noguera Louzada

**Affiliations:** 1 Universidade Federal do Rio de Janeiro Faculdade de Medicina Department of Ophthalmology and Otorhinolaryngology Rio de Janeiro RJ Brazil Department of Ophthalmology and Otorhinolaryngology, Faculdade de Medicina, Universidade Federal do Rio de Janeiro, Rio de Janeiro, RJ, Brazil.; 2 Universidade Cidade de São Paulo Faculdade de Medicina São Paulo SP Brazil Faculdade de Medicina, Universidade Cidade de São Paulo, São Paulo, SP, Brazil.; 3 Wills Eye Hospital Glaucoma Research Center Philadelphia PA USA Glaucoma Research Center, Wills Eye Hospital, Philadelphia, PA, USA.; 4 Pontifícia Universidade Católica do Paraná Faculdade de Medicina Curitiba PR Brazil Faculdade de Medicina, Pontifícia Universidade Católica do Paraná, Curitiba, PR, Brazil.; 5 Instituto de Educação Médica Faculdade de Medicina Angra dos Reis RJ Brazil Faculdade de Medicina, Instituto de Educação Médica, Angra dos Reis, RJ, Brazil.; 6 Universidade de Brasília Faculdade de Medicina Brasília DF Brazil Faculdade de Medicina, Universidade de Brasília, Brasília, DF, Brazil.; 7 Universidade Estadual do Rio Grande do Norte Faculdade de Medicina Mossoró RN Brazil Faculdade de Medicina, Universidade Estadual do Rio Grande do Norte, Mossoró, RN, Brazil.; 8 Universidade de São Paulo Faculdade de Medicina Divisão de Oftalmologia e Laboratório de Investigação em Oftalmologia São Paulo SP Brazil Divisão de Oftalmologia e Laboratório de Investigação em Oftalmologia (LIM-33), Faculdade de Medicina, Universidade de São Paulo, São Paulo, SP, Brazil.

**Keywords:** Cataract, Lens, intraocular, Phacoemulsification, Workflow

## Abstract

**Introduction:**

Cataracts are the leading cause of reversible blindness worldwide, with age-related cataracts being the most common type. With advancements in digital workflows, new alternative surgical processes aim to enhance efficiency and patient outcomes.

**Objective:**

This study aimed to evaluate the effectiveness of digital *versus* manual workflows for cataract surgery through a systematic review and meta-analysis focusing on preoperative assessment time, surgery planning time, intraoperative duration, and transcription frequency.

**Methods:**

The study was performed in accordance with PRISMA guidelines and identified relevant studies published until July 2024 in the PubMed, Embase, Web of Science, and Cochrane databases.

**Results:**

Digital workflows significantly reduced preoperative assessment and intraoperative times for astigmatic cataracts (mean difference (MD)=80.94 s, p<0.01; MD=107.13 s, p=0, respectively) and planning times (MD=130.52 s, p=0.43). Additionally, digital workflows decreased transcription requirements for conventional and post-refractive cataracts. Heterogeneity was notable, especially in the preoperative assessments (I^2^ >90%).

**Conclusion:**

The findings suggest that digital workflows for cataract surgery improve efficiency; however, further large-scale, long-term studies are required to assess the broader applicability and cost-effectiveness of these workflows. Digitalization has the potential to streamline the surgical management of cataracts and enhance patient outcomes.

**Prospero database registration:**

ID CRD42024590552.

## INTRODUCTION

Cataracts, characterized by the opacification of the crystalline lens or its surrounding capsule, are the leading cause of reversible blindness worldwide.^(1,2)^ Age-related cataracts, which typically begin between the ages of 45 and 50 years, remain the most common type of cataract in adults.^(3)^ In 2023, the World Health Organization estimated that 94 million people were visually impaired owing to cataracts, which continue to be the leading cause of blindness in middle- and low-income countries.^(4,5)^ The global population of individuals aged ≥80 years is projected to reach 265 million by the mid-2030s, emphasizing the growing burden of age-related cataracts.^(6)^ By the late-2050s, 61 million individuals are expected to be blind, 474 million will experience moderate-to-severe vision impairment, and 360 million will have mild vision impairment.^(7)^ Thus, cataracts will remain a prominent public health challenge owing to the aging population.^(8, 9)^

Cataracts often require surgical interventions as they progress and interfere with daily activities.^(10–12)^ Although the use of refractive glasses can offer temporary relief in the early stages of the disease, cataract surgery remains the primary treatment modality, as it leads to the correction of visual function and improvement of quality of life.^(13)^ Cataract surgery, one of the most frequently performed procedures worldwide, has seen considerable advancements owing to improvements in surgical techniques and technologies.^(14–16)^ This intervention requires detailed preoperative planning to ensure the selection of an intraocular lens (IOL) with an appropriate refractive power for optimizing postoperative visual outcomes. The preoperative procedures include a thorough ocular examination and precise ocular biometry, including measurements of axial length, anterior chamber depth, lens thickness, corneal white-to-white dimensions, and keratometry measurements.^(3,17–19)^

With the increasing adoption of toric IOL implants, which require additional time and precision, surgeons are increasingly seeking optimized workflows and advanced tools for accurate IOL power calculations. Although manual workflows are commonly used, they are often inefficient and prone to errors.^(20)^ Reviewing the diagnostic results typically requires access to paper charts or separate digital image management systems that are not integrated with those used for lens or toric power calculations.^(19)^ Therefore, planning a single surgery can involve the collection of data from multiple sources-such as optical biometry reports, online calculators for toric and spherical IOL power, corneal topographic images, optical coherence tomographic scans, and medical records.^(21)^ This fragmented approach not only increases the risk of errors but also introduces significant inefficiencies.

A comprehensive digital health solution, distinguished by its ability to optimize cataract surgery management through a digital workflow, has emerged.^(20)^ It facilitates an advanced integration between diagnostic devices and surgical equipment via a cloud-based infrastructure, which not only centralizes and streamlines access to clinical and operational data but also enhances the accuracy and efficiency of the surgical process.^(18,20,22,23)^ Digital systems unify diagnostic and planning data on a single platform. This minimizes transcription errors and data mismatches, ensures precise measurements for selecting the appropriate IOL, and optimizes surgical outcomes. By enhancing accuracy, these systems improve patient safety and reduce the risk of complications associated with preoperative planning inaccuracies.^(15,17)^

Digital workflows support surgical training in addition to improving efficiency. Although these systems do not typically offer full surgical simulations, they allow for the visualization of real-time diagnostic images. This supports improved preoperative planning by enabling surgeons to view and analyze patient data on a unified platform.^(16)^ These resources enable trainees to develop skills and gain a deeper understanding of surgical planning, thereby facilitating performance tracking and feedback.

Although there is no consensus regarding the best workflow for cataract surgery, trials comparing these methods are emerging. However, no meta-analysis has compared digital and manual workflows.

## OBJECTIVE

To evaluate the effectiveness of digital *versus* manual workflows for cataract surgery, this systematic review and meta-analysis examined the impact of both processes on preoperative assessment, time efficiency, and intraoperative duration.

## METHODS

The present study was conducted in accordance with the Cochrane Collaboration Handbook for Systematic Reviews of Interventions and the Preferred Reporting Items for Systematic Reviews and Meta-Analysis (PRISMA) guidelines.^(24,25)^

### Eligibility criteria

There were no restrictions on the publication date, status, or language. The following studies were included: (1) randomized clinical trials (RCTs) or non-RCTs (2) that included patients who underwent cataract surgery; (3) compared digital *versus* manual workflows for cataract surgery; and (4) reported the clinical outcomes of interest—preoperative assessment, time efficiency, and intraoperative duration.

### Data source and search strategy

We systematically searched the PubMed, Embase, Web of Science, and Cochrane databases, with the search updated most recently in July 2024. The complete search strategy was as follows: ("Cataract" OR "Intraocular implants" OR "Intraocular Lens" OR "phacoemulsification" OR "phaco") AND ("Workflow" OR "planning software" OR "time-saving" OR "time saving" OR "surgical planning" OR "surgery planning" OR "digitalization" OR "preoperative planning"). All retrieved records were independently reviewed by two authors, who made decisions regarding full-text retrieval by consensus. Both authors then examined the full texts and decided on the inclusion or exclusion of studies based on the predefined criteria. References from eligible papers and reports on systematic reviews were reviewed to identify the relevant studies. Conference abstracts and prospective trials were searched to identify additional studies of interest.

### Data extraction

Two authors independently extracted data and gathered key information from each study, including publication year; study design; sample size; patient age; cataract type; and relevant outcomes such as preoperative assessment time, surgery planning duration, intraoperative time, and transcription frequency. For studies with missing summary statistics, we used imputation methods in accordance with the Cochrane guidelines and standardized any non-uniform units. The results were organized and visually displayed using spreadsheets to support systematic synthesis and comparisons across studies.

### Risk of bias assessment

The risk of bias in nonrandomized studies was evaluated using the Risk of Bias in Non-Randomized Studies—of Interventions (ROBINS-I) tool.^(26)^ Based on specific domain evaluations, each study was classified as having a "low risk," "moderate risk," or "serious risk." Two independent authors conducted the assessments, and any disagreements were resolved by consensus following discussions regarding discrepancies.

### Statistical analysis

Odds ratios (ORs) with 95% confidence intervals (95%CIs) were used to compare the treatment effects for categorical endpoints, whereas mean differences (MDs) were used to compare the treatment effects for continuous outcomes. Statistical significance was set at p<0.05. Heterogeneity among studies was assessed using Cochran's Q test, the I^2^ test, and the τ^2^ test. An I^2^ value greater than 50% indicated high statistical heterogeneity, prompting the use of a random-effects model for these analyses. A random-effects model was used for all analyses. Statistical analyses were conducted using the R software, version 4.0.1 (R Foundation for Statistical Computing, Vienna, Austria).

### Sensitivity analysis

A leave-one-out (LOO) sensitivity analysis was used to assess the impact of each study on the overall pooled estimate. This analysis involved sequentially excluding one study at a time to evaluate how its omission influenced the pooled analysis results.

### Quality assessment

Two independent authors assessed the risk of bias in the included non-RCTs using the Cochrane tool for assessing the risk of bias in non-RCTs (ROBINS-I). Any disagreements were resolved by consensus.

## RESULTS

**Figure 1:**
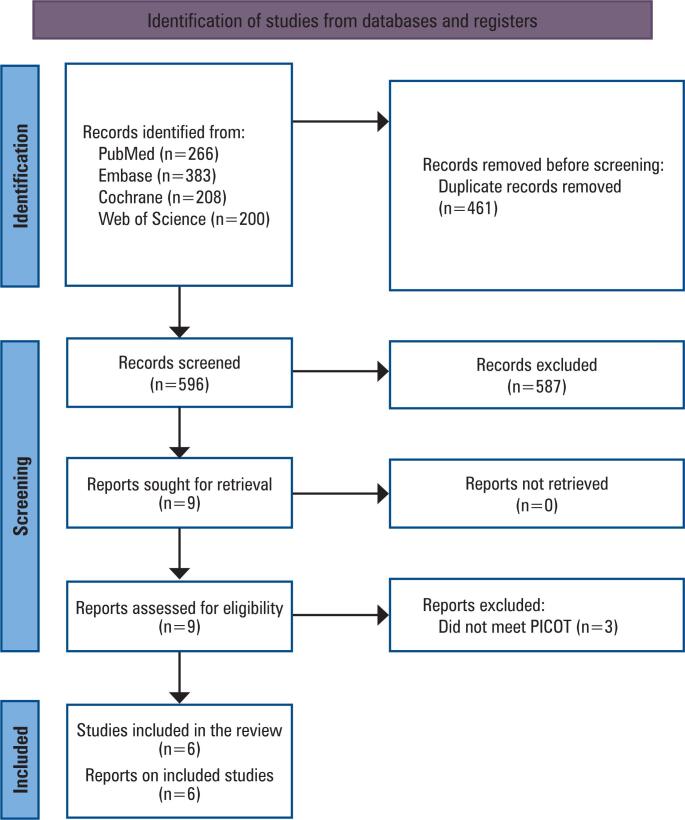
PRISMA flow diagram describing the study screening and selection process

PRISMA: Preferred Reporting Items for Systematic reviews and Meta-Analyses; PICOT: Population, Intervention, Comparison, Outcome, and Timeframe.

A systematic literature search yielded 1,057 articles. After removing duplicate records and studies that met the exclusion criteria based on title/abstract review, six observational prospective studies were included (Figure 1). Among the patients included in these six studies, 472 (54%) underwent surgery using the digital workflow and 401 (46%) underwent surgery using the conventional workflow. The characteristics of the included studies are presented in table 1.

**Table 1:** Baseline characteristics

Study	Country	Design	Population digital/conventional workflow	Eye digital/conventional workflow	Mean age (year)	Male: Female	Cataract type-Astigmatic	Cataract type-Astigmatic	Cataract type-Conventional
Rombold et al.(27) 2024	Germany	Prospective	227/203	227/203	NA	NA	403	NA	NA
Brunner et al.(20) 2022	Germany	Prospective	24/24	24/24	60 ± 9.8	9/15	24	NA	NA
Gujral et al.(18) 2021	USA	Prospective	40/40	40/40	69.33 ± 13.3	19/21	20	9	11
Zavodni et al.(22) 2023	USA	Prospective	55/55	55/55	NA	NA	10	10	10
Shetty et al.(29) 2024	India	Prospective	66/19	66/19	NA	NA	NA	NA	NA
Russell et al.(28) 2024	Australia	Prospective	30/30	60/60	74.75 ± 7.14	30/30	60	0	0

NA: not applicable.

### Pooled analysis of all studies

#### Preoperative assessment times

**Figure 2:**
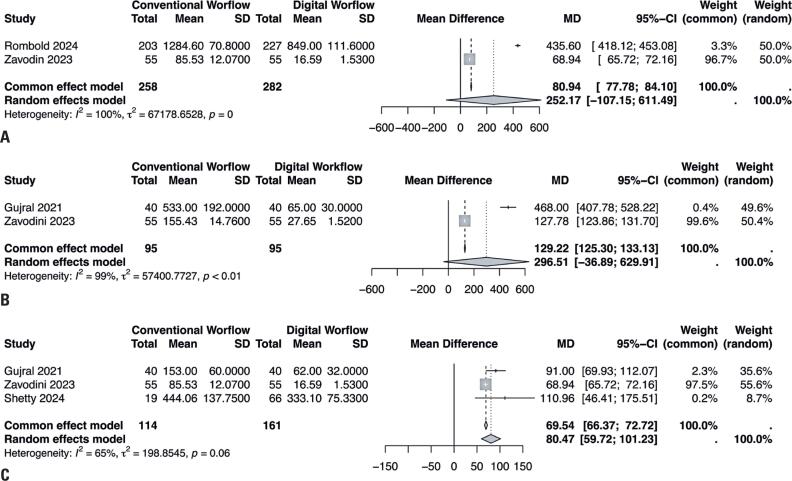
(A) Forest plot presenting the preoperative assessment times of the digital *versus* conventional workflows for astigmatic cataracts. (B) Forest plot presenting the preoperative assessment times of the digital *versus* conventional workflows for post-refractive cataracts. (C) Forest plot presenting the preoperative assessment times of the digital *versus* conventional workflows for conventional cataracts

MD: mean difference; SD: standard deviation; 95%CI: 95% confidence interval.

We compared the conventional and digital workflows and analyzed the preoperative assessment times, measured in seconds, for patients with astigmatic, conventional, and post-refractive cataracts.^(18,20,22,27–29)^ Two studies reported the outcomes in 540 eyes with astigmatic cataracts. Our analysis showed no statistically significant differences in the preoperative assessment times (MD=252.17, 95%CI= [−107.15 to 611.49], I^2^=100%; Figure 2A).

Similarly, two studies analyzed 190 eyes with post-refractive cataracts and found no significant differences between the workflows (MD=296.51, 95%CI= [−36.89 to 629.91], I^2^=99%; Figure 2B).

In contrast, significant differences were observed in preoperative assessment times for 275 eyes with conventional cataracts in three studies (MD=80.47, 95%CI= [59.72 to 101.23], I^2^=65%; Figure 2C).

### Preoperative surgery planning

**Figure 3:**
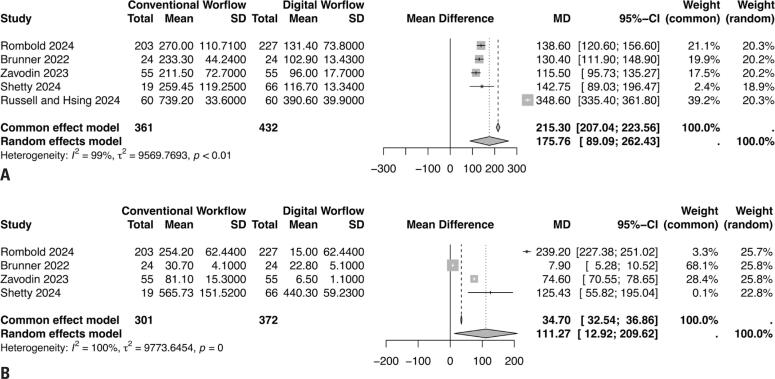
(A) Forest plot presenting preoperative surgery planning of digital *versus* conventional workflows for astigmatic cataracts. (B) Forest plot presenting intraoperative procedures of digital *versus* conventional workflows for astigmatic cataracts

MD: mean difference; SD: standard deviation; 95%CI: 95% confidence interval.

Six studies compared preoperative surgical planning in patients with astigmatic cataracts. A total of 793 eyes were analyzed, and the analysis revealed a statistically significant difference favoring the digital workflow over the conventional approach (MD=175.76, 95%CI= [89.09 to 262.43], I^2^=99%; Figure 3A).

### Intraoperative procedures

Intraoperative procedures for astigmatic cataracts were compared between the conventional and digital workflows. Three studies reported this outcome, and 673 eyes were analyzed. The analysis revealed a statistically significant difference favoring the digital workflow (MD=111.27, 95%CI= [11.92 to 209.62], I^2^=100%; Figure 3B).

### Number of transcriptions

**Figure 4:**
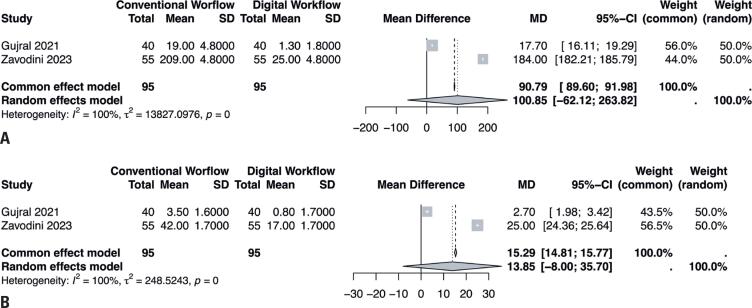
(A) Forest plot presenting the transcription count of digital *versus* conventional for post-refractive cataracts. (B) Forest plot presenting the transcription count of digital *versus* conventional for conventional cataracts

The number of transcriptions required during the cataract surgery workflows was assessed for patients who underwent conventional and digital procedures. Two studies reported data from 190 eyes with post-refractive cataracts and showed no significant differences between the workflows (MD=100.85, 95%CI= [−62.12 to 263.82], I^2^=100%; Figure 4A).

Similar findings were reported for the number of transcriptions in 190 eyes with conventional cataracts (MD=13.85, 95%CI= [−8.00 to 35.70], I^2^=100%; Figure 4B). Significant heterogeneity was noted across all analyses, which led to the use of LOO plots to detect the outlier studies.

### Risk of bias assessment

**Figure 5:**
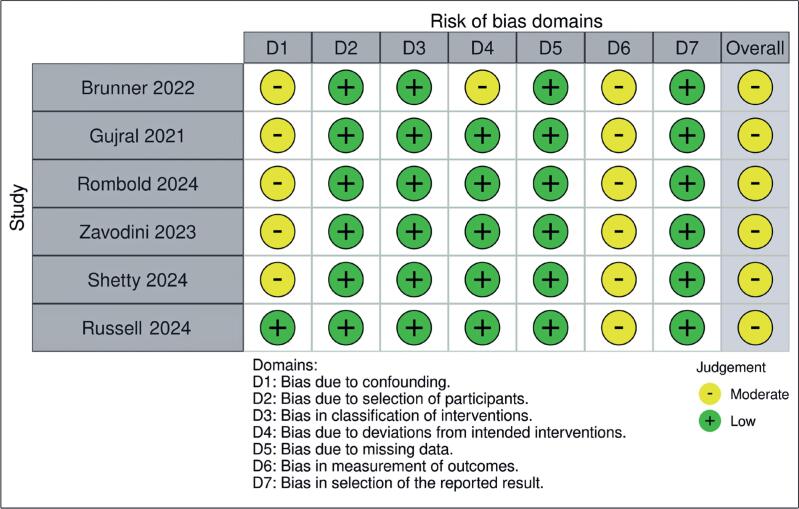
Risk of bias assessment of non-RCTs using the ROBINS-I tool Non-RCT, nonrandomized controlled trial; ROBINS-I, Risk of Bias in Non-Randomized Studies-of Interventions

MD: mean difference; SD: standard deviation; 95%CI: 95% confidence interval.

Figure 5 presents the results of the evaluation of the risk of bias for each study. Three studies had a moderate risk of bias in the confounding factor domain. One study raised concerns about bias in participant selection, and another study showed a moderate risk of bias owing to missing data. The remaining domains in each study were rated as "low risk," indicating that most studies had a low overall risk of bias across domains.

### Sensitivity analysis

Given the significant heterogeneity observed in several outcomes in our meta-analysis, we used the LOO technique to identify potential outliers. For the preoperative assessment times in patients with conventional cataracts, the LOO analysis revealed a notable reduction in heterogeneity when the studies by Gujral et al. and Zavodni et al. were excluded.^(18,22)^ Statistically significant differences were preserved even after the exclusion of the studies by Gujral et al. and Zavodni et al. (MD=77.08, 95%CI= [44.53 to 109.62], p<0.01; I^2^=38% and MD=92.96, 95%CI= [72.72 to 113.21], p<0.01; I^2^=0%, respectively).

For the preoperative surgery-planning outcome, the LOO analysis indicated that heterogeneity significantly decreased only when the study by Russell and Hsing was excluded. A statistically significant difference was maintained even after the exclusion of the aforementioned study (MD=129.42, 95%CI= [117.31 to 141.53], p<0.01; I^2^=5%).

The LOO analysis did not identify any study whose exclusion meaningfully reduced heterogeneity in the case of intraoperative procedures for patients with astigmatic cataracts.

## DISCUSSION

This systematic review and meta-analysis of six studies compared the use of 472 (54.07%) digital cataract workflows with 401 manual (conventional) workflows (45.93%). The results revealed significant efficacy of digital workflows for cataract surgery, with the following key findings separately compared in patients with astigmatic, conventional, and post-refractive cataracts: (1) preoperative assessment time was significantly shorter for digital workflows for all types of cataracts; (2) digital workflows compared to manual workflows required fewer transcriptions for conventional and post-refractive cataracts (data relating to astigmatic cataracts were not provided in the studies analyzed); and (3) digital workflows reduced planning and intraoperative times pertaining to astigmatic cataract procedures (other types were not analyzed in the selected studies).

Notably, no articles contradicting the superior results of the digital workflow were found. Although its implementation presents challenges such as a high initial cost, the literature supports its efficiency and states that it even provides advantages such as resource efficiency, according to Shetty et al.^(29)^

Our meta-analysis found that for astigmatic cataracts: (1) the preoperative assessment time for the digital workflow was significantly shorter than that for the conventional workflow (MD=80.94 s; p<0.01 for the common-effect model; MD=252.17s; p<0.01 for the random-effects model); (2) the digital workflow reduced planning time compared to the conventional workflow (MD=130.52s; p=0.43 for the common-effect model; MD=130.52s; p=0.43 for the random-effects model); and (3) regarding, the digital workflow was associated with a significantly shorter intraoperative time than the conventional workflow (MD=34.61s; p=0 for the common-effect model; MD=107.13s; p=0 for the random-effects model).

Our results showed superior efficacy of the digital workflow for conventional cataracts: (1) the preoperative assessment time of the digital workflow was shorter than that of the conventional workflow (MD=54.54s; p<0.01 for the common-effect model; MD=71.01s; p<0.01 for the random-effects model); (2) the digital workflow requires fewer transcriptions than the conventional workflow (MD=15.29 transcriptions; p=0 for the common-effect model; MD=13.85 transcriptions; p=0 for random effects model).

Similarly, for patients with post-refractive cataracts: (1) the digital workflow was notably faster than the conventional workflow (MD=126.88s; p<0.01 for the common-effect model; MD=295.35s; p<0.01 for the random-effects model); (2) the digital workflow required significantly fewer transcriptions (MD=90.79 transcriptions; p=0 for the common-effect model; MD=100.85 transcriptions; p=0).

Regarding the finding that the preoperative assessment time was much shorter in cases where the digital workflow was used, Shetty et al. stated that integrating the digital workflow into existing electronic medical record workflows significantly reduced the mean time for preoperative measurements by 25.3% (p=0.006).^(29)^ Finally, Russel et al. concluded that for patients with astigmatism, the median presurgical planning time was 6.51±0.65 min for the digital workflow and 12.32±0.56 min for the manual workflow (p<0.001).

According to the results of the present study, the digital workflow requires fewer transcripts. Shetty et al. recorded fewer data fields in a digital workflow (p<0.0001), implying that the digital workflow requires fewer data entries, which is in line with the finding of fewer transcriptions noted in the present study.^(29)^

Despite the absence of direct data supporting the claim that digital workflows require fewer transcriptions, the present study contributes to the discussion on the superiority of digital workflows in various ways. For instance, See et al. have emphasized that digital workflows enhance surgical planning by utilizing advanced imaging techniques and improving IOL calculations, which could reduce manual data handling and, consequently, reduce transcription requirements.^(30)^ Xia et al. have stated that digital documentation can enhance communication among surgical teams, potentially leading to more efficient data transfer and reduced redundancy in record-keeping.^(31)^ Brandsdorfer et al. performed a literature review focusing on astigmatism management through digital workflows, presenting findings that indicated enhanced measurement accuracy and indirectly supported a more efficient process that could minimize transcription needs.^(32)^ Although none of these studies explicitly quantified transcription count reductions, their findings collectively state the effectiveness of digital workflows, hinting at a favorable shift toward modern practices in cataract surgery.

Few data are available regarding the evidence that digital workflow reduces planning and intraoperative times. In the study by Mayer et al., the mean overall time required to perform the surgery was significantly shorter in the digital group (727.2±198.4s *versus* 1110.0±382.2s; p<0.001).^(33)^ Although Russell and Hsing did not record the intraoperative time, they did record the difference in the total workflow time between the manual workflow (13.49 ± 0.47 min) and the digital workflow (6.93±0.57 min).

The advantages of using digital workflows extend beyond cataract surgery, and they are emerging as a growing trend in other surgical specialties. In several studies,^(30–32)^ such as studies on restorative and implant dentistry, digital navigation systems have been associated with reduced procedure times, improved surgical efficiency, reduced human errors, increased precision in movement, and even a difference in peri-implant crestal bone loss.^(31)^ These findings suggest that the benefits of digitalization extend across various surgical fields.^(34–36)^

The transition to digital workflows poses significant challenges, particularly in low-resource settings. Although the high initial costs can be a barrier, these systems offer the potential for long-term savings through error reduction and improved resource efficiency, ultimately enhancing patient outcomes. Financial constraints and the steep learning curve for mastering digital workflows often discourage clinicians from abandoning the traditional methods. Ongoing training is crucial to fully leverage the accuracy and efficiency of digital technologies. Although regular software and hardware updates increase the cost and complexity of digital workflows, they are essential for ensuring compatibility with new materials and fabrication techniques.^(37)^

This study had certain limitations. None of the analyzed articles mentioned the patient follow-up period, with Brunner et al. only mentioning a 3-month follow-up period without providing any further details. Thus, the benefits and potential malfunctions of digital workflows could not be analyzed. Most studies have focused on specific cataract types (*e.g*., astigmatic, conventional, and post-refractive), which may limit the generalizability of the findings to other cataract types or broader patient populations. However, planning and intraoperative times have not been reported for conventional post-refractive cataracts. Finally, significant heterogeneity was noted in preoperative assessment times (I^2^ ranging from 93% to 100%) and the number of transcriptions across studies (I^2^=100%). This variability can affect the overall interpretation and applicability of the results.

The literature comparing digital and manual workflows in cataract surgery is relatively new, resulting in the inclusion of few references. The scarcity of studies makes it challenging to conduct point-by-point comparisons of the results obtained in our meta-analysis for each type of cataract.

Digital workflows are essential tools that are more efficient than the conventional methods. However, further research with larger sample sizes, more extended follow-up periods, and standardized measures is needed to confirm these findings and fully understand the benefits and limitations of digital *versus* conventional workflows for cataract surgery.

## CONCLUSION

The findings of the present study suggest that digital workflows for cataract surgery have the potential to significantly improve the efficiency and reduce the procedural time of cataract surgery, particularly in patients with astigmatic cataracts.

However, this study had several limitations that should be considered. The retrospective nature of most included studies and the heterogeneity in patient populations and surgical techniques may have influenced the results. Additionally, the lack of long-term follow-up data limits our understanding of the long-term impact of digital workflows on patient outcomes.

Future research should prioritize large-scale randomized controlled trials with standardized protocols and longer follow-up periods to deliver more robust evidence. Additionally, exploring the cost-effectiveness of digital workflows compared to traditional methods would be beneficial.

Addressing these limitations and conducting further research will enhance our understanding of the benefits and challenges of digital workflows for cataract surgery. This knowledge will ultimately improve patient outcomes and optimize the surgical process.
